# Metagenomic Approach Deciphers the Role of Community Composition of Mycobiome Structured by *Bacillus velezensis* VB7 and *Trichoderma koningiopsis* TK in Tomato Rhizosphere to Suppress Root-Knot Nematode Infecting Tomato

**DOI:** 10.3390/microorganisms11102467

**Published:** 2023-09-30

**Authors:** Vinothini Kamalanathan, Nakkeeran Sevugapperumal, Saranya Nallusamy, Suhail Ashraf, Kumanan Kailasam, Mohd Afzal

**Affiliations:** 1 Department of Plant Pathology, Centre for Plant Protection Studies, Tamil Nadu Agricultural University, Coimbatore 641003, Tamil Nadu, India; vinokjc19@gmail.com; 2 Department of Plant Molecular Biology and Bioinformatics, Centre for Plant Molecular Biology and Biotechnology, Tamil Nadu Agricultural University, Coimbatore 641003, Tamil Nadu, India; saranya.n@tnau.ac.in; 3 Department of Plant Biotechnology, Tamil Nadu Agricultural University, Coimbatore 641003, Tamil Nadu, India; suhailashraf9906@gmail.com; 4 Department of Horticulture, Agricultural College & Research Institute, Kudumiyanmalai, TNAU, Pudukottai 622104, Tamil Nadu, India; kumanan@tnau.ac.in; 5 Department of Chemistry, College of Science, King Saud University, Riyadh 11451, Saudi Arabia; maslam1@ksu.edu.sa

**Keywords:** tomato, *Meloidogyne incognita*, *Bacillus velezensis* VB7, *Trichoderma koningiopsis* TK, mycobiome

## Abstract

The soil microbiome is crucial for maintaining the sustainability of the agricultural environment. Concerning the role of diverse mycobiomes and their abundance toward the suppression of root-knot nematode (RKN) infection in vegetable crops, our understanding is unclear. To unveil this issue, we examined the fungal microbiome in tomato rhizosphere augmented with bioagents challenged against RKN at taxonomic and functional levels. Composition of the mycobiome in tomato rhizosphere treated with *Bacillus velezensis* VB7 and *Trichoderma koningiopsis* TK differed significantly from the infected tomato rhizosphere. The abundance and diversity of fungal species, however, were significantly higher in the combined treatments of bioagents than for individual treatments. Fungal microbiome diversity was negatively correlated in the RKN-associated soil. Network analysis of the fungal biome indicated a larger and complex network of fungal biome diversity in bioagent-treated soil than in nematode-associated tomato rhizosphere. The diversity index represented by that challenging the RKN by drenching with consortia of *B. velezensis* VB7 and *T. koningiopsis* TK, or applying them individually, constituted the maximum abundance and richness of the mycobiome compared to the untreated control. Thus, the increased diverse nature and relative abundance of the mycobiome in tomato rhizosphere was mediated through the application of either *T. koningiopsis* TK or *B. velezensis* VB7, individually or as a consortium comprising both fungal and bacterial antagonists, which facilitated engineering the community composition of fungal bioagents. This in turn inhibited the infestation of RKN in tomato. It would be interesting to explore further the possibility of combined applications of *B. velezensis* VB7 and *T. koningiopsis* TK to manage root-knot nematodes as an integrated approach for managing plant parasitic nematodes at the field level.

## 1. Introduction

Fungi are a ubiquitous and extremely diversified group of organisms that play a significant role in ecological and biogeochemical processes as plant symbionts and decomposers in soil [1,2]. The elements of the environment have a significant impact on the structure and composition of fungal communities. The abundance of fungal communities flourish in a given habitat and their relative competition is determined by the interactions of plants and microbes, abiotic factors, and the availability and quality of organic substrates [3]. The abundance of fungal diversity is responsible for plant health and can be documented by utilizing genetic markers combined with high-throughput sequencing. In order to understand the functions of fungi in terrestrial ecosystems, it is now possible to identify the factors that sustain or threaten fungal diversity, and to investigate the relationships between the diversity of communities during abiotic and biotic interactions [4]. The specialized nature and tropism of the microorganisms in the rhizosphere affect the number and variety of the operational taxonomic units (OTUs). In addition, the composition and stability of belowground microbial communities are influenced by a variety of factors, including farming practices and aboveground vegetation [5]. As a consequence of the influence of a variety of factors, plant parasitic nematodes (PPN) have a crucial role in reducing the productivity of tomato.

PPNs are an imminent threat to the cultivation of crops [6]. RKN, *Meloidogyne* spp., are obligate plant parasites, with the ability to infect all vascular plants, both under protected agriculture, in greenhouses, or in the field. In the recent past, infection by nematodes in crop plants caused a crop loss of 14%, accounting for an annual loss of 125 billion US$ [7]. In these circumstances, management of RKN remains a major challenge because it is an obligate endoparasite with a broad host range, high reproductive rate, and short generation time [8]. PPNs are managed by nematicidal molecules; however, the use of nematicidal molecules pollutes the environment, groundwater, and human health [9]. After realizing the impact of nematicidal molecules, it is imperative to develop an ecofriendly approach to curb RKN through the alteration of the microbial community in the rhizosphere, which can be mediated through effective bioagents with nematicidal action. Based on this, perusal of the literature revealed that plant growth-promoting rhizobacteria (PGPR) could curb the growth and survival of PPNs by producing lytic enzymes. Further, they colonize the rhizosphere and plant roots, enhance plant growth and development, and impart protection against plant diseases through the secretion of antimicrobial peptides [10]. Earlier evidence emphasizes that *Bacillus* spp. and *Trichoderma* spp. are of major significance, as they have nematicidal properties [11,12]. Besides, several other research findings also emphasize that the combined use of different biocontrol agents complements the synergistic interactions and thus quench nematode proliferation and infection [13]. However, the specific combinations and their effectiveness may vary depending on the target nematode species, crop, and environmental conditions [14].

Multiple investigations by different researchers revealed that introducing *Bacillus* spp. and *Trichoderma* spp. enhanced crop productivity by changing the diversity in the rhizosphere microbial community for different crops [15]. An increased abundance in microbial taxa with beneficial metabolic activity contributes to increased crop productivity [16]. Expanding knowledge on the plant microbiome has sparked interest in biological control of plant diseases, and has emphasized the need to explore for alternatives to chemicals for managing the disease. Insights into changes in microbial composition associated with RKN parasitism have also opened new avenues for the management of the fungal nematode complex [17]. Similarly, the application of bioagents may also alter the physiology of roots or have an impact on the diversity of the rhizosphere’s mycobiome, which could have indirect effects on taxa that serve as growth promoters or biocontrol agents. However, research concerning the diversity of the mycobiome altered in the presence of RKN or RKN challenged with bioagents is only in an infancy stage. Considering this, in our previous study with the same experimental design, we exclusively described the bacterial community in both bioagent-treated and untreated tomato rhizosphere that influences the diversity of different bacterial communities in reducing the RKN infestation. 

The main objective of the present study is to investigate soil microbial diversity and how it relates to the presence of biocontrol agents in soil when compared to soil infected with root-knot nematodes (RKN). Additionally, we explore identifying the specific microbiome that may be associated with influencing RKN infection. Thus, by using metagenomic sequencing to control RKN in tomato, we conducted research focused on analyzing mycobiome abundance in tomato rhizosphere infected with RKN and RKN challenged with fungal and bacterial antagonists. Because fungi are ubiquitous and have mutualistic relations with other beneficial organisms, the diversity of the fungal community in the rhizosphere plays a significant role in reducing the RKN infection through various mechanisms and maintaining a healthy soil ecosystem.

## 2. Materials and Methods

### 2.1. Preparation of Nematode Inoculum

Eggs were collected from severely infected galled tomato roots. To separate the eggs from the gelatinous matrix, the roots were chopped into pieces of 1–2 cm, placed in a 500 mL plastic container, filled with 1.5% chlorine solution, and shaken vigorously for 3 min. The suspension was then rinsed four times with running water through a 250 mm sieve; the eggs were collected on a sieve with a mesh size of 20 mm. After 4 days of incubation at 28 ± 2 °C, the hatched second-stage juveniles (J2) were collected from the egg suspension using a modified Baermann dish. The hatched second-stage juveniles (J2) were collected and utilized for the experiments.

### 2.2. Preparation of Liquid Formulations of B. velezensis VB7 and T. koningiopsis TK

The bacterial inoculum of *B. velezensis* VB7 (MW301630) used for this experiment was prepared as per the standard protocol [18]. The single colony of bacteria was inoculated into LB broth, which was then maintained in an orbital shaker at 150 rpm, at room temperature (28 ± 2 °C) for 48 h to ensure uniform bacterial growth. The culture broth was mixed with 1% glycerol (10 mL), 1% tween 20 (10 mL), and 1% polyvinylpyrrolidone g). The mixture was incubated in an orbital shaker at 200 rpm for 5 min to ensure uniform mixing. The bacterial suspension of the liquid formulation was adjusted to 5 × 10^8^ CFU mL^−1^.

The fungal antagonists *T. koningiopsis* TK (KX 555650) were cultured in potato dextrose broth and incubated in an orbital shaker at 150 rpm at room temperature (28 ± 2 °C) for 120 h. Later, the liquid biomass with mycelial and conidial suspension was mixed with 1% glycerol (10 mL), Tween 20 (10 mL), and poly vinyl pyrrolidone—40,000 mL wt. (10 g), each separately. The resultant mixture was kept in an orbital shaker at 200 rpm for 5 min to ensure the uniform homogenization. Then, the formulation was standardized to obtain 3 × 10^8^ cfu/mL. The liquid formulation was stored at 5 °C for further study [19].

### 2.3. Bioefficacy of the Liquid Formulation of B. velezensis VB7 and T. koningiopsis TK against M. incognita Infestation in Greenhouse Condition

To assess the efficacy of the liquid formulation of *B. velezensis* VB7 (MW301630) and *T. koningiopsis* TK (KX 555650), pot culture experiments were conducted in a greenhouse at the Department of Plant Pathology, TNAU, Coimbatore, Tamil Nadu, India, by maintaining three biological replicates for each treatment. Tomato hybrid Sivam was used throughout the study. Antagonistic bacteria *B. velezensis* VB7 and *T. koningiopsis* TK, were tested under pot culture to assess their efficacy against RKN in tomato. Bioagents were treated either individually or as combined formulations with both bioagents either challenged with or without RKN. The pots were filled with 7 kg of sterilized potting mixture containing red soil: sand: cow dung manure @ 1:1:1 *w*/*w*/*w*. To it, 20-day-old tomato seedlings were transplanted. After transplanting the tomato seedlings, a hole was made by inserting 4 mm thick iron rods to a depth of 2 cm around the tomato plants. The rods were removed after 2 days and juvenile suspension @ one juvenile/gram of soil was poured into holes and covered with sterile soil. Simultaneously, the soil was drenched with a liquid formulation of *B. velezensis* VB7 @1% suspensions (5 × 10^8^ cfu/mL) [18] and 1% *T. koningiopsis* TK @ 3 × 10^8^ cfu/mL [19]. Later, formulation of the respective antagonists was delivered to soil at 15, 30, 45, and 60 days after the first application. Three replications were maintained for each treatment. Five pots were maintained for each replication. Five seedlings were maintained in each pot. Observation of the root-knot nematode incidence was recorded at 75 days after application based on the root galling index GI (0–5), as suggested by [20]. The plant height and fruit yield per plant were recorded periodically.

### 2.4. Collection of Samples

A field trial in tomato was established at Thondamuthur in Coimbatore province, Tamil Nadu, India (GPS coordinates: 10.5484° N 76.2857° E) for the management of RKN, which was endemic for the infection of RKN. Twenty-day-old Sivam tomato hybrid was planted in the field with row-to-row spacing of 2 ft and plant-to-plant spacing of 2 ft. Rhizosphere soils were drenched until saturation with liquid formulations of 1% *T. koningiopsis* TK (3 × 10^8^ cfu/g) and *B. velezensis* VB7 (5 × 10^8^ cfu/mL) comprising six different treatments. The treatment details included: T1—*B. velezensis* VB7 + RKN, T2—*B. velezensis* VB7 alone, T3—*T. koningiopsis* + RKN, T4—*T. konigiopsis* alone, T5—*B. velezensis* VB7 + *T. koningiopsis* + RKN, and T6—untreated control. Three biological replicates were maintained for each treatment. Each replicate was laid over an area of 40 m^2^. To analyze the impact of different treatments on the population diversity of the mycobiome in the rhizosphere through metagenomics approach, soil samples were collected at 35 days after transplanting, since it coincided with the peak vegetative phase. Collected samples were stored in sterile aluminum foil, immediately placed in an ice box, transported to the laboratory, and stored at −80 °C until processing.

### 2.5. PCR Amplification of 18S rRNA

Using the Power Soil DNA Isolation Kit after 35 days of planting, metagenomic DNA was separately extracted from the rhizosphere soil that was collected from different treatments comprising T1—*B. velezensis* VB7 + RKN, T2—*B. velezensis* VB7 alone, T3—*T. koningiopsis* TK + RKN, T4—*T. konigiopsis* TK alone, T5—*B. velezensis* VB7 + *T. koningiopsis* TK + RKN, and T6—untreated control in RKN infected tomato fields. Quantitative and qualitative analysis of DNA was performed by the Nanodrop method followed by agarose gel electrophoresis. A total of 50 ng DNA from each sample was subjected to PCR amplification using 18 s rRNA gene for fungal ribosomal operon region-specific primers KYO—FP (5′-ATAGAGGAAGTAAAAGTCGTAA-3′) and LE31-RP (5′-ATGGTCCGTGTTTCAAGAC-3′). The amplicons were confirmed by Agarose (1%) with EtBr gel electrophoresis. The PCR products were purified using 1.6x Ampure XP beads (Beckmann Coulter, Brea, CA, USA).

### 2.6. Library Preparation and Sequencing of DNA Product

A total of ~50 ng from each amplicon DNA was end-repaired (NEBnext ultra II end repair kit,), and cleaned with 1x AmPure beads. Barcoding adapter ligation (BCA) was performed with NEB blunt/TA ligase and cleaned with 1x AmPure beads. Qubit quantified adapter-ligated DNA samples were barcoded using PCR reactions and pooled at equimolar concentration; end-repair was performed using NEBnext ultra II end repair kit (New England Biolabs, Ipswich, MA, USA) and cleaned. Adapter ligation (AMX) was performed for 15 min using NEB blunt/TA ligase (New England Biolabs, MA, USA). The library mix was cleaned using Ampure beads and finally eluted in 15 μL of elution buffer. 

Sequencing was performed for both prokaryotic and eukaryotic organisms through the Oxford Nanopore sequencing method using MinION flow cell R9.4 (FLO-MIN106). Nanopore raw reads (‘fast5′ format) were base-called (‘fastq5′ format) and demultiplexed using Guppy1 v2.3.4.

### 2.7. Processing of Sequencing Data and Taxonomic Profiling

Sequencing data were processed with the Guppy v2.3.4 tool kit for base calling the sequencing data to generate pass read. The adapter and barcode sequences were trimmed using the Porechop tool. The reads were filtered by size using SeqKit software ver. 0.10.0 and the average Phred quality score was assessed using the SILVA database. A comprehensive taxonomic analysis of microbial communities was performed on the processed reads from each set. To conduct the diversity analysis, the obtained rRNA reads were imported into Qiime2 in the Single End Fastq Manifest Phred33 input format. The readings were deduplicated and then grouped into operational taxonomic units (OTUs) based on 97% similarity [21]. BLAST with QIIME (categorize–consensus–search) was used to classify representative sequences using percent identity 0.97 against the SILVA full-length 16S database. The long-read amplicons data were sequenced by using Nanopore MinION and validated against SILVA databases to determine their proper classification. The relative microbial abundances were determined to categorize the microbial community for each individual sample according to their taxonomic profile (Kingdom, Phylum, Class, Order, Family, Genus, Species-level) in a stacked bar chart. Singletons and sequences classified as mitochondria, chloroplast, archaea, and unassigned sequences were removed. Only the top 20 fungal OTUs were used for the analysis.

### 2.8. Diversity Index Analysis

The microbial diversity analysis was estimated by calculating the alpha and beta diversity indexes by using the obtained OTU cluster. The alpha diversity was carried out using the evenness vector, Jaccard, observed features vector [22], and Shannon vector [23] algorithms to determine the diversity and richness of the microbial community within the sample, whereas the beta diversity represents the diversity between different samples using the Bray–Curtis dissimilarity statistic [24]. Samples were compared by applying the wide set of multivariate statistical tools in PERMANOVA together with visualization tools. According to Zhang and collaborators, OTU comparisons were performed using the Venn diagram package [25]. All of these indices for our samples were calculated using QIIME (Version 1.7.0, http://qiime.org/1.7.0/ accessed on 15 May 2023). Principal coordinates (PCoA) plots were generated to determine the community structure using QIIME1 version 1.9.1 [26]. Statistical analyses were carried out using R statistical software version 4.2.3 with a diverse set of subprograms [27]. All significance tests were two-sided, and *p* values < 0.05 were considered statistically significant. Gephi 0.10.1 was used to construct the co-occurrences coefficient network by using the mean average of OTUs for each sample.

### 2.9. Taxonomic Abundance of the Microbial Population through Cluster Heatmap

According to the abundance of information on microbial communities at the taxonomic level, a heatmap was constructed by clustering similar communities of each sample to determine the frequency of microbial communities.

### 2.10. Venn Diagram

Venn diagram was constructed to determine the relationship between the fungal communities that reside in both treated and untreated soil for comparing at genus and species level for the combined applications of bioagents. It was performed using the Muthor program and then submitted to VENNY (http://bioinfogp.cnb.csic.es/tools/venny/index.html; accessed on 15 May 2023) to show the shared and unique OTUs [15].

### 2.11. Statistical Analysis

All of the experiments were analyzed independently. The treatment means were compared by applying Duncan’s multiple range test (DMRT). The package used for analysis was SPSS version 16.0., developed by IBM Corporation, and interpreted using a critical difference at *p* = 0.05.

## 3. Results

### 3.1. Effect of a Liquid Formulation of B. velezensis VB7 and T. koningiopsis TK against Root-Knot Nematode (M. incognita) in Tomato under Greenhouse Conditions

**Figure 1:**
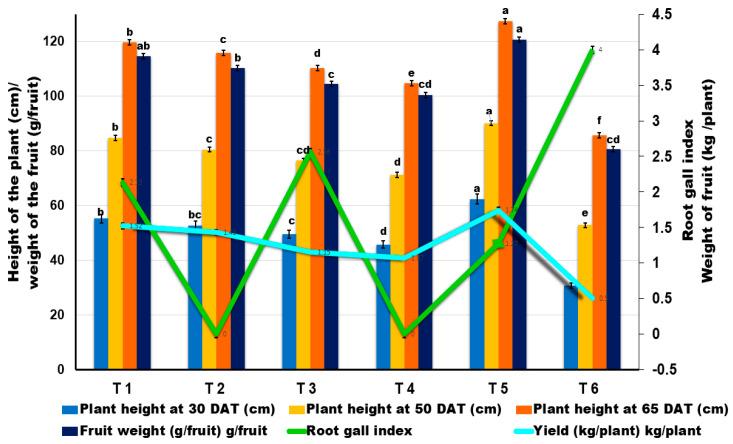
Effect of liquid formulation of *B. velezensis* VB7 and *T. koningiopsis* TK against root-knot nematode (*M. incognita*) in tomato under greenhouse conditions. Error bars indicate the standard deviation obtained from three replicates. Analysis of variance was performed through DMRT. Means followed by different letters indicate significant differences (*p* < 0.05; *n* = 5) between treatments. (T1—*B. velezensis* VB7 (1%) + RKN, T2—*B. velezensis*, T3—*T. koningiopsis* TK + RKN, T4—*T. koningiopsis*,T5—*B. velezensis,* (1%) + *T. koningiopsis* (1%) + RKN, T6—RKN (*M. incognita*)).

Soil drenching with *B. velezensis* VB7 (1%) along with *T. koningiopsis* TK (1%) challenged with RKN in tomato plants on the 15th, 30th, and 45th day after planting was effective in suppressing the incidence of RKN. Further, it also promoted more plant growth than the individual application of neither bacterial nor fungal antagonists used in the study. Combined soil application with *B. velezensis* VB7 (1%) and *T. koningiopsis* TK (1%) reduced the gall index to 1.27 with an average fruit weight of 120.75 g/fruit and with a mean yield of 2.36 kg/plant. It was followed by soil drenching with *B. velezensis* VB7 (1%) challenged with RKN, giving a gall index of 2.13, fruit weight of 114.60 g/fruit with a mean fruit yield of 1.92 kg/plant. Similarly, the tomato plants treated with *T. koningiopsis* TK challenged against RKN expressed a gall index of 2.56 with a fruit weight of 104.58 g/fruit and a mean yield of 1.45 kg/plant. However, the higher RKN incidence of fourth grade gall index was recorded in the untreated control. Similarly, there was no significant difference in the fruit weight/plant and fruit yield/plant with respect to the consortia comprising individual applications of bioagents, either in the presence of RKN or without RKN. Thus, the results indicated the ability for the consortia-based application of liquid formulation of *B. velezensis* VB7 and *T. koningiopsis* TK to effectively manage RKN infestation in tomato plants in greenhouse cultivation (Figure 1, and Supplementary Information Table S1).

### 3.2. Identification of OTU and Taxonomic Annotation for Fungal Communities

A total of 36,479 raw reads with an average of 6080 reads were generated from the Illumina Miseq sequencing of the six samples. After quality control, 33,278 clean reads with an average of 5546 reads remained. The quality-filtered reads were further size filtered to obtain the classified OUT to retain the 500–600 bp sequences of the ITS region. Totally, 3740 sizes of filtered reads ranging from 200 to 1386 were identified after processing of QC filtered reads. A total of 31 OUTs were identified as classified read. Among these, 16 (30.77%) for *B. velezensis* VB7 + *T. koningiopsis* TK + RKN, 6 (12.25%) for *B. velezensis* VB7 + RKN, 2 (25%) for RKN alone, 5 (29.5%) for *T. koningiopsis* TK alone, 7 (28%) for *T. koningiopsis* TK + RKN, and 5 (17.25%) for *B. velezensis* VB7 alone RKN were used for further analysis.

### 3.3. Analysis of the Microbial Community’s Composition

#### Relative Abundance

**Figure 2:**
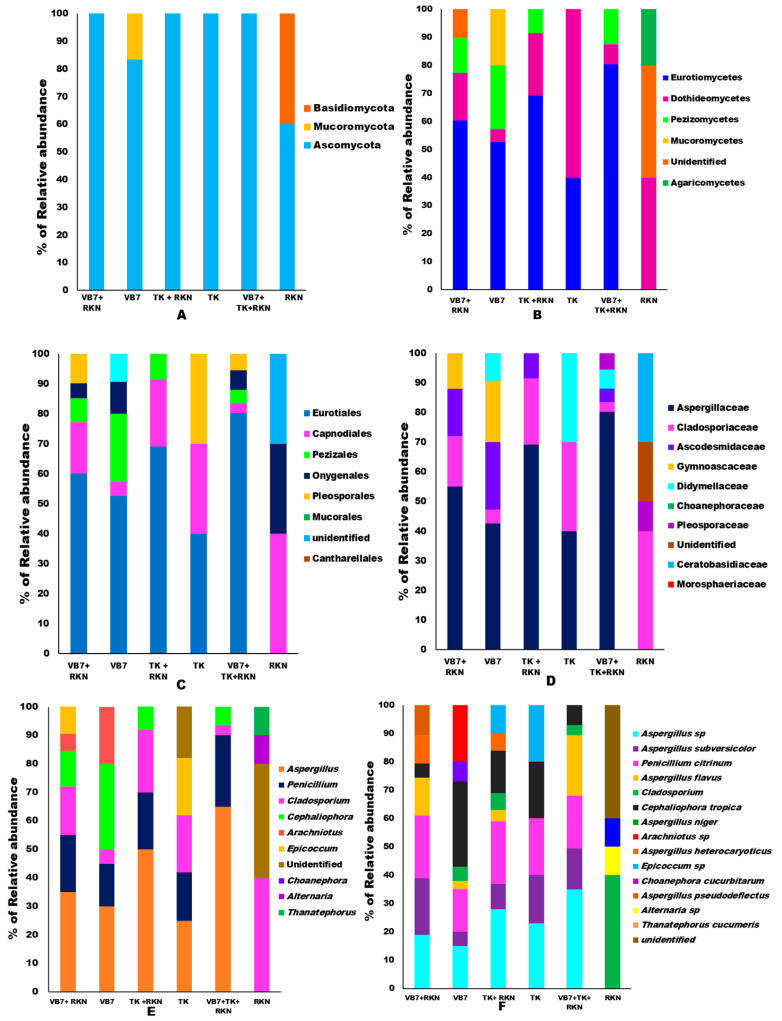
Relative abundances of fungal communities with respect to different treatments: (**A**) fungal phylum; (**B**) fungal classes; (**C**) fungal orders; (**D**) fungal families; (**E**) fungal genera; (**F**) fungal species. (VB7 + RKN *= B. velezensis* VB7 + root-knot nematode, VB7 *= B. velezensis* VB7 alone, TK + RKN = *T. koningiopsis* TK + root-knot nematode, TK *= T. koningiopsis* TK alone, VB7 + TK + RKN = *B. velezensis VB7+ T. koningiopsis* TK + root-knot nematode, RKN *=* root-knot nematode (*M. incognita*).

According to the microbial classification, 14 phyla, 20 classes, 22 orders, 24 families, 28 genera, and 34 species of different fungal communities were identified in all six different tomato rhizosphere soils during the present investigation. The taxonomic annotation of each sample was grouped to each level to determine the proportion of relative abundance of each sample at different taxonomic classification levels. The abundance of Phylum for each taxa level and each sample has been represented in a staked bar chart and illustrated as s (Figure 2A–E). Ascomycota, Basidiomycota, and Mucoromycota were the three predominant fungal Phyla found in all samples. Among them, Ascomycota (93%) had the highest composition and was more prominent than Basidiomycota (5%) and Mucoromycota (2.43%) (Supplementary Information, Figure S1). Phylum Ascomycota constituted the maximum relative abundance of 100% in tomato rhizosphere soils treated with *B. velezensis* VB7 + RKN, *T. koningiopsis* TK + RKN, *T. koningiopsis* TK alone, and *B. velezensis* VB7 + *T. koningiopsis* TK + RKN-treated soils. It was followed by 83.33% in soil applied with *B. velezensis* VB7 alone and 60% in RKN-infested soil. The second Phylum, Basidiomycota, was observed only in untreated RKN soil, with the relative abundance of 40%, whereas, the third Phylum of Mucoromycota had the lowest relative abundance, 16.66%, in tomato rhizosphere soil treated with *B. velezensis* VB7 alone (Figure 2A). As a consequence, the application of bioagents in combination had the maximum impact on the establishment of the Ascomycota population in tomato rhizosphere soil, and might therefore be responsible for the suppression of RKN.

Investigation of fungal classes present in different rhizosphere soil samples revealed the occurrence of Agaricomycetes, Eurotiomycetes, Dothideomycetes, Mucoromycetes, and Pezizomycetes. Among them, the classes of Eurotiomycetes (50%) were predominant in all the rhizosphere soils, followed by Dothideomycetes (29%), Pezizomycetes (5%), Agaricomycetes (5%), Mucoromycetes (3%) and 7% of unidentified classes (Supplementary Information, Figure S2). Comparative analysis of relative abundance indicated that Ascomycetes had the highest mean relative abundance, 80.25%, in tomato rhizosphere soil treated with *B. velezensis* VB7 + *T. koningiopsis* TK + RKN. However, the relative abundance was 69.25% in tomato rhizosphere soil treated with *T. koningiopsis* TK in the presence of RKN, as against 40% in *T. koningiopsis* TK drenched tomato rhizosphere soil. Whereas, 60.25% of Eurotiomycetes abundance was recorded in the tomato rhizosphere soil drenched with *B. velezensis* VB7 + RKN, but the abundance level was 52.66% in soils applied with *B. velezensis* VB7. However, it is intriguing to note that it was not present in RKN-infested soil. Dothideomycetes was ranked second in terms of abundance level with 60.42% in *T. koningiopsis* TK treated-soils without RKN infestation, followed by 40.37% in RKN-infested soil. However, drenching of *T. koningiopsis* TK onto the tomato rhizosphere infested with RKN had greater abundance (22.89%) compared to *B. velezensis* VB7 + RKN-infested rhizosphere soil (17.25%). The lowest relative abundance of 4.65% was observed in *B. velezensis* VB7-applied soils. Analysis of Agaricomycetes revealed that the relative abundance was 20.87% in RKN-infested soil. The population of Pezizomycetes was exclusively observed in *B. velezensis* VB7 + RKN with a greater abundance of 22.51% (Figure 2B). Thus, the metagenomics data revealed that microbial communities in tomato rhizospheres were enhanced through the synergistic interaction of compatible bioagents and rhizobiome, which facilitated reduction in RKN infestation.

The fungal orders, including Pleosporales, Eurotiales, Pezizales, Mucorales, Capnodiales, Cantharellales, and Onygenales were commonly associated in all rhizosphere soils regardless of treatment. Among them, Eurotiales (49%) was the predominant community in soils, followed by Pleosporales (24.39%), Pezizales (5%), Capnodiales (5%), and Cantharellales (5%). Mucoroales (3%) and Onygenales (2%), however, had the lowest composition (Supplementary Information, Figure S3). The soil drenched with *B. velezensis* VB7 followed by *T. koningiopsis* TK in RKN-challenged rhizosphere had a greater relative abundance of 80.25% of Eurotiales. However, the RKN-infested soil treated with *B. velezensis* VB7 had 60.25% abundance of Eurotiales as contrasted with 52.75% in *B. velezensis* VB7 without RKN association. On the contrary, the application of *T. koningiopsis* TK in the presence of RKN had a greater proportion, 69.52%, when compared to *T. koningiopsis* TK, which had only 40%. The Eurotiales communities were negligible in untreated RKN-infected soil. The Pleosporales order was present in soils treated with bioagents alone except in *T. koningiopsis* TK. The tomato rhizosphere soil amended with *T. koningiopsis* TK alone had an increased abundance of 30.52% followed by *B. velezensis* VB7 + RKN with 15.14% abundance, as against 10.66% for the relative abundance in *B. velezensis* VB7. Order Pezizales was detected only in soil drenched with *B. velezensis* VB7 alone with a greater proportion of 22.5%, as against 8.4% in *B. velezensis* VB7 + RKN-treated tomato rhizosphere soils. However, the tomato rhizosphere drenched with a combination of *B. velezensis* VB7 + *T. koningiopsis* TK + RKN had only 4.59%. The abundance of Capnodiales was 40.75% in RKN-infested soil followed by 22.25% in *T. koningiopsis* TK + RKN, whereas the same was only 4.56% in tomato rhizosphere soil drenched with *B. velezensis* VB7. Furthermore, 30.23% of unidentified fungal communities were solely dominant in RKN-associated soil (Figure 2C).

The comparative analysis on the relative abundances of different fungal families in all soil samples comprised Aspergillaceae, Didymellaceae, Ceratobasidiaceae, Pleosporaceae, Gymnoascaceae, Choanephoracea, Ascodesmidaceae, Morosphaeriaceae, and Cladosporiaceae. Among them, Aspergillaceae (49.56%) and Didymellaceae (20.21%) were dominant in all samples (Supplementary Information, Figure S4). The relative abundance of Aspergillaceae population was predominant in the tomato rhizosphere amended with *T. koningiopsis* TK when compared to *B. velezensis* VB7. RKN-infested tomato soil drenched with *B. velezensis* VB7 followed by *T. koningiopsis* TK had 80.25% abundance of Aspergillaceae. The abundance of Aspergillaceae was 69.25% in soils applied with *T. koningiopsis* TK + RKN-treated rhizosphere soil as against 40.56% in *T. koningiopsis* TK alone. On the contrary, the abundance of the Aspergillaceae population in *B. velezensis* VB7-treated soil with infestation of RKN had 55.34% of abundance. However, it was only 42.35% in the rhizosphere soils drenched with *B. velezensis* VB7 in the absence of RKN. It was not present in the untreated control soil. The presence of the Didymellaceae population was only observed in rhizosphere soil amended with individual applications of bioagents without RKN infestation and combined application of bioagents in RKN-infected soils. The greater proportion of Didymellaceae population was observed in *T. koningiopsis* TK-treated soil with 30.14% abundance, followed by 10.85% in *B. velezensis* VB7 and 6.52% in *B. velezensis* VB7 + *T. koningiopsis* TK + RKN drenched rhizosphere soils. The RKN-infected soil had 40.74% abundance of Cladosporiaceae, followed by 30.77% and 22.66% in *T. koningiopsis* TK-applied soil and *B. velezensis* VB7-drenched soils, respectively. Interestingly, it was noticed that fungi belonging to Ceratobasidiaceae and Morosphaeriaceae inhabited solely in soil associated with RKN with abundance of 30% and 10%, respectively. While the relative abundance of Gymnoascaceae and Choanephoraceae communities were observed to be present only in *B. velezensis* VB7-treated soil. The abundance of Gymnoascaceae in *B. velezensis* VB7 had 20.67% and 12.75% in RKN-associated soil drenched with *B. velezensis* VB7. However, Choanephoraceae was associated only in healthy rhizosphere soil drenched with *B. velezensis* VB7 (10.45%). Overall, 9.78% of unidentified organisms were detected in soil samples, with 30.38% of RA (Figure 2D). Aspergillaceae population increased in response to bioagent application, while RKN-infested soil influenced the proliferation of Cladosporiaceae, Ceratobasidaceae, and Pleosporaceae communities. The abundance of Didymellaceae, however, was induced by the application of *T. koningiopsis* TK.

The common fungal genera Aspergillus, Penicillium, Cladosporium, Cephaliophora, Arachniotus, Epicoccum, Choanephora, Alternaria, and Thanatephorus were observed in tomato rhizosphere soil. Among them, Aspergillus (46.23%), Epicoccum (12.5%), and Cephaliophora (5.34%) genera were dominant in all the soil samples (Supplementary Information Figure S5). The relative abundance of Aspergillus genus occupied the maximum percentage of 65.43% in RKN-associated soil drenched with *B. velezensis* VB7, followed by *T. koningiopsis* TK. However, the abundance of Aspergillus population was 50.18% in tomato rhizosphere treated with *T. koningiopsis* TK in the presence of RKN. But the abundance was only 25.94% in tomato rhizosphere soil drenched with *T. koningiopsis* TK. However, the soil drenched with *B. velezensis* VB7 + RKN was 35.87% and 30.65% in *B. velezensis* VB7 without RKN infestation. The abundance of Penicillium was 25.67% and differed significantly from combined application of bioagents from *B. velezensis* VB7 + T. koningiopsis TK + RKN. It was followed by soils of tomato rhizosphere drenched with *B. velezensis* VB7 + RKN and T. koningiopsis TK + RKN-infested soil. Analysis on the abundance of the genus Penicillium revealed that the relative abundance in the rhizosphere soil drenched with *T. koningiopsis* TK was 17.85% compared to 15.43% in tomato rhizosphere soils drenched with *B. velezensis* VB7 alone, while the Epicoccum genus resides only in soil amended with bioagents and infested with RKN. Abundance of the genus Epicoccum in tomato rhizosphere soil treated with *T. koningiopsis* TK + RKN was 20.57%, whereas 20% of abundance was noticed in soil treated with *T. koningiopsis* TK, compared to 10.67% in soil treated with *B. velezensis* VB7 alone. The tomato RKN-infected rhizosphere soil drenched with *T. koningiopsis* TK was abundant in the population of Cladosporium genera, accounting for 22.67% as against 20.24% in *T. koningiopsis* TK. Abundance of Cladosporium in *B. velezensis* VB7 + RKN-drenched soil was 17.54% compared to 5.45% in *B. velezensis* VB7-drenched soils. The RKN-infested soil had constituted a greater proportion of Cladosporium (40.17%). However, the maximum relative abundance of 10.67% of the genus Thanatephorus was recorded in RKN-infested soil (10.67%). The genus Alternaria was present only in soil infested with RKN and its abundance was 10%. The unidentified fungal communities were observed to be greatest in the RKN-infested soil (30.86%). The introduced bioagents were highly compatible with Aspergillus and Penicillium genus, which increased the abundance diversity and reduced the RKN infestation. Similarly, drenching of *B. velezensis* VB7 to tomato rhizosphere increased the abundance of the Cephaliophora genus (Figure 2E).

The diversity of fungal species observed in various rhizosphere soils include *Aspergillus* sp., *A. subversicolor*, *Penicillium citrinum*, *A. flavus*, *Cladosporium* sp., *Cephaliophora tropica*, *A. niger*, *Arachniotus* sp. *A. heterocaryoticus*, *Epicoccum* sp., *Choanephora cucurbitarum*, *A. pseudodeflectus*, *Alternaria* sp., and *Thanatephorus cucumeris* (Supplementary Information, Figure S6). Among them, *T. cucumeris* (22.5%), *Aspergillus* sp. (20.57%), *A. subversicolor* (7.5%) and *A. flavus* (7.5%) were predominant in tomato rhizosphere. The soil treated with *T. koningiopsis* TK + *B. velezensis* VB7 + RKN had a greater percentage of *Aspergillus* spp. (35.65%), followed by *A. flavus* (21.70%), *P. citrinum* (18.65%), *A. subversicolor* (14.56%), and *Cladosporium* sp. (3.68%), while the rhizosphere soil applied with *T. koningiopsis* TK in RKN-associated soil had the maximum abundance value of 23.75% with respect to *Aspergillus* sp. It was followed by *P. citrinum* (20.75%), *A. subversicolor* (17.98%), *A. flavus* (4.52%), *Cephaliophora tropica* (12.5%), and *Cladosporium* sp. (6.25%). In contrast, the rhizosphere soil drenched with *B. velezensis* VB7 alone had the relative abundance of 30.64% pertaining to *Cephaliophora tropica*, and was followed by 15.36% abundance value in the soils drenched with *T. koningiopsis* TK + RKN. *A. pseudodeflectus* was solely present only in *T. koningiopsis* TK + RKN soil. The relative abundance of *Epicoccum* sp. observed in the soil treated with *T. koningiopsis* TK and *B. velezensis* VB7-treated soil was 20.33% and 10.18%, respectively. Further, RKN soil had a major proportion of unidentified species (40.0%), whereas *T. cucumeris* and *Alternaria* spp. occupied a lesser proportion (10%) in rhizosphere (Figure 2F).

### 3.4. Taxonomic Abundance of Fungal Population Analyzed through a Cluster Heatmap

**Figure 3:**
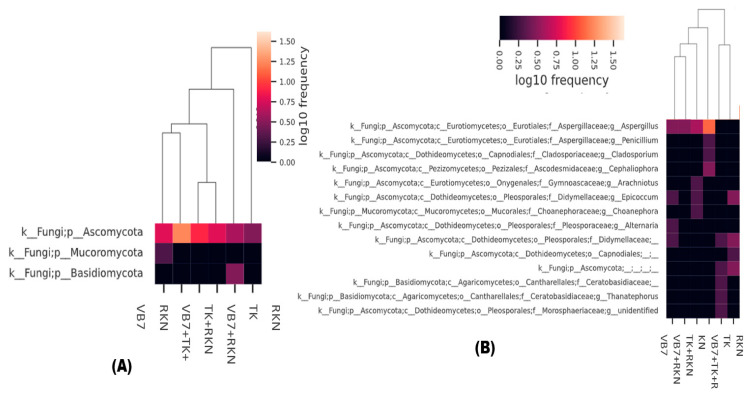
(**A**) Cluster heatmap for the distribution of fungal phyla and (**B**) fungal species. (VB7 + RKN = *B. velezensis* VB7 + root-knot nematode, VB7 *= B. velezensis* VB7 alone, TK + RKN = *T. koningiopsis* TK + root-knot nematode, TK = *T. koningiopsis* TK alone, VB7 + TK + RKN = *B. velezensis* VB7+ *T. koningiopsis* TK+ root-knot nematode, RKN = root-knot nematode (*M. incognita*)).

According to the abundance of information on microbial communities at the taxonomic level, a heatmap was constructed by clustering similar communities of the fungal population in each sample to determine the frequency of microbial communities. The dominant fungal genera of Ascomycota, Basidiomycota, and Mucoromycota had different frequencies, and varied with respect to different treatments. Tomato rhizosphere soil treated with *B. velezensis* VB7 followed by *T. koningiopsis* TK in RKN-associated soil had the highest frequency (1.5%) of Ascomycota phylum, while the other soil samples had a lower frequency level. Distribution frequency of Basidiomycota was 0.5 in RKN-infested soil. However, Mucoromycota was present only in VB7 soil samples with the lowest frequency level of 0.25% (Figure 3A). Tomato rhizosphere soil drenched with *B. velezensis* VB7 + *T. koningiopsis* TK, challenged against RKN, had 1.25% frequency level of the *Aspergillus* genus. It was followed by *Penicillium, Cladosporium*, and *Cephaliophora*. However, the population frequency of *Thanatephorus* and *Alternaria* populations was lower in RKN-infested soil (Figure 3B).

### 3.5. Comparison of OTUs in Different Treatments

**Figure 4:**
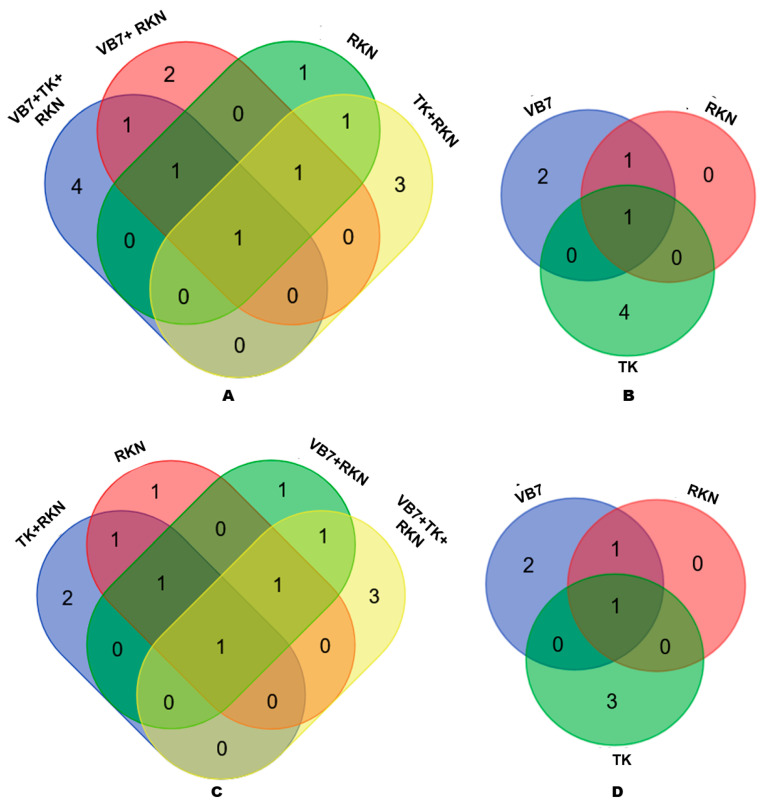
(**A**) Comparison of OTUs at the genus level in combined application. (**B**) Comparison of OTUs at genus level in individual applications. (**C**) Comparison of OTUs at the species level in combined applications. (**D**) Comparison of OTUs at the species level in the individual application. (VB7 + RKN = *B. velezensis* VB7 + root-knot nematode, VB7 = *B. velezensis VB7* alone, TK + RKN = *T. koningiopsis* TK + root-knot nematode, TK = *T. koningiopsis* TK alone, VB7 + TK + RKN = *B. velezensis* VB7 + *T. koningiopsis* TK + root-knot nematode, RKN = root-knot nematode (*M. incognita*)).

A total of 31 classified OTUs were obtained through high-throughput sequencing. A number of OTUs distribution varied between individual and combined applications. The number of OTUs present in soils treated with bioagents associated with RKN or without RKN and control (untreated) were compared at genus and species levels to determine the presence of unique organisms. At the genus level, only 1 OTU was shared by all four samples, and the unique OTUs for *B. velezensis* VB7 + RKN, *T. koningiopsis* TK + RKN, *B. velezensis* VB7 + *T. koningiopsis* TK challenged against RKN and control (untreated) soil were 3, 2, 4, and 1 at genus level, respectively, whereas 1 OTU was common for all four of the abovementioned soils at the species level with unique OTUs of 2, 1, 3, and 1, respectively (Figure 4A,B). Similarly, the distribution of OTUs in the individual soil application of *B. velezensis* VB7, *T. koningiopsis* TK, and control (untreated) were compared at genus and species levels. A total of 1 OTU at the genus and species level was commonly distributed in all combined application treatments. It was followed by similar OTUs of 4, 2, and 0 at the genus level and 3, 2, and 0 OTUs at the species level in tomato rhizosphere soil drenched with *B. velezensis* VB7, *T. koningiopsis* TK, and control (Figure 4C,D).

### 3.6. Diversity of Fungal Communities in Different Rhizosphere Soils

#### 3.6.1. Alpha Diversity Indexes

**Figure 5:**
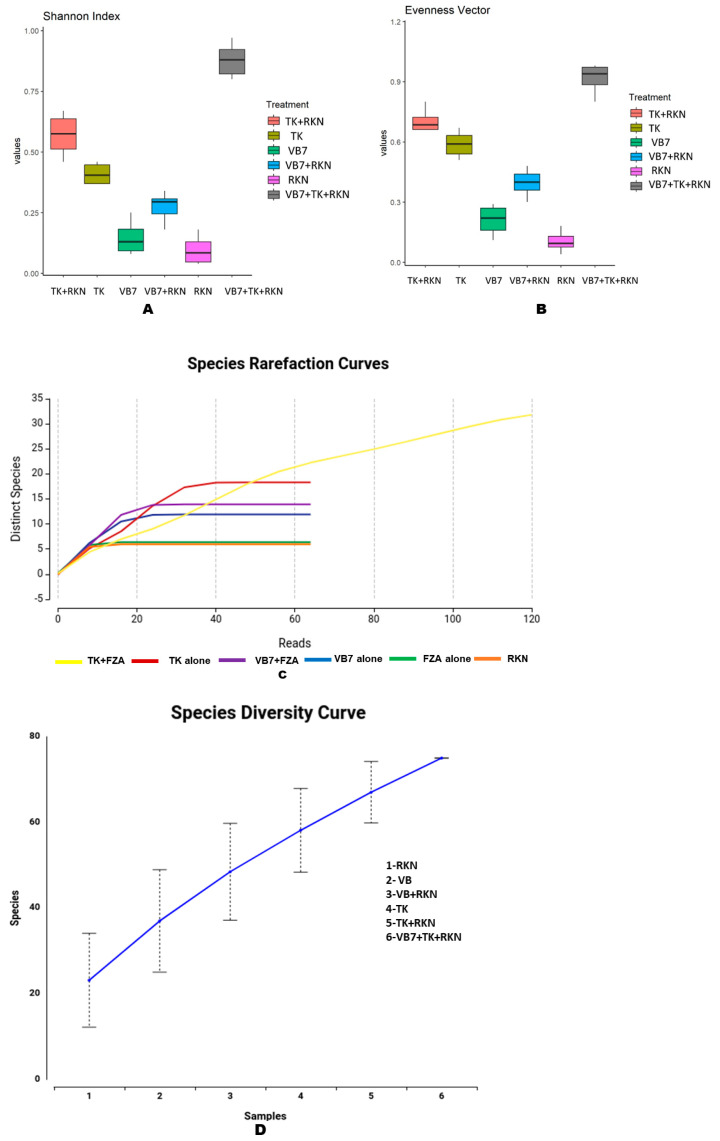
Alpha diversity index for fungal communities in different treatments: (**A**) Shannon index; (**B**) evenness vector algorithm; (**C**) refraction curve; (**D**) species diversity curve. (VB7 + RKN = *B. velezensis* VB7 + root-knot nematode, VB7 = *B. velezensis* VB7 alone, TK + RKN = *T. koningiopsis* TK + root-knot nematode, TK = *T. koningiopsis* TK alone, VB7 + TK + RKN = *B. velezensis* VB7 + *T. koningiopsis* TK + root-knot nematode, RKN = root-knot nematode (*M. incognita*).

Alpha diversity was used to analyze the richness and diversity of microbial communities present in the soil. The rhizosphere soil pertaining to *B. velezensis* VB7 + *T. koningiopsis* TK challenged against RKN had the maximum Shannon index in all taxonomic levels from Phylum to Species, followed by *T. koningiopsis* TK challenged against RKN. The fungal communities in RKN-infested soil and *B. velezensis* VB7 alone-drenched soil had almost similar levels of richness with lower indexes when compared to other samples. The diversity of the mycobiome in *T. koningiopsis* TK + RKN soil and *B. velezensis* VB7 soil were not found to differ significantly from each other (Figure 5A). Similarly, the evenness vector algorithm indicated that tomato rhizosphere soil drenched with *B. velezensis* VB7 + *T. koningiopsis* TK + RKN harbored the higher species diversity with greater abundance of fungal communities when compared to others. The soil drenched with *T. koningiopsis* TK had the maximum abundance of fungal species (evenness) compared *B. velezensis* VB7-treated soil with a lower level of microbial diversity and lesser homogeneity of organisms (Figure 5B). The refraction curve showed a significant increase in fungal species and indicated that the combined application of bioagents had the maximum fungal communities with more diverse fungal species than the rhizosphere soils amended with individual bioagents (Figure 5C). The species diversity curve analysis revealed that the number of fungal species in soil varied with respect to each treatment. The RKN-infested tomato rhizosphere soil treated with the consortia of *B. velezensis* VB7 + *T. koningiopsis* TK had greater species diversity when compared with *T. koningiopsis* TK- or *B. velezensis* VB7-treated soil without RKN. However, a lower species diversity was observed in untreated RKN-infected soil. Hence, the diversity and richness of fungal population was not found to differ significantly in tomato rhizosphere soils drenched with combined application of *B. velezensis* VB7 + *T. koningiopsis* TK (Figure 5D).

#### 3.6.2. Beta Diversity Indexes

**Figure 6:**
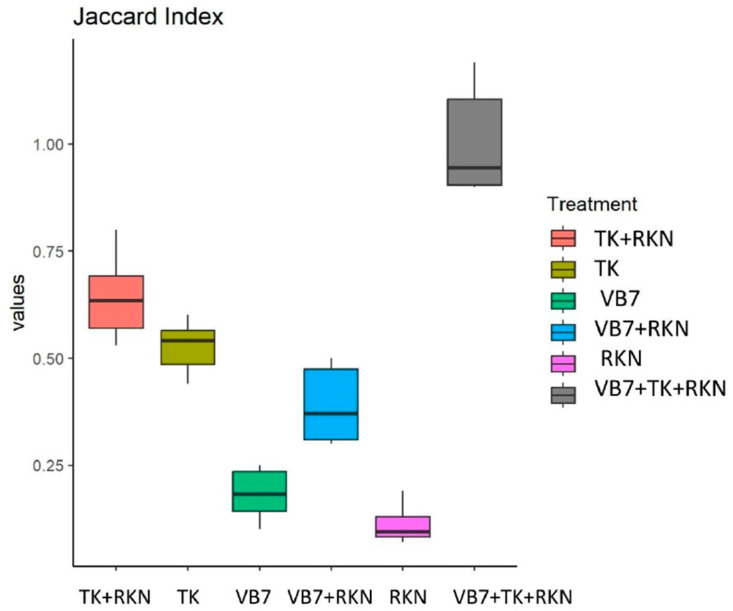
Beta diversity index for fungal communities in different treatments: Jaccard index. (VB7 + RKN = *B. velezensis* VB7 + root-knot nematode, VB7 = *B. velezensis* VB7 alone, TK + RKN = *T. koningiopsis* TK + root-knot nematode, TK = *T. koningiopsis* TK alone, VB7 + TK + RKN = *B. velezensis* VB7+ *T. koningiopsis* TK + root-knot nematode, RKN = root-knot nematode (*M. incognita*)).

The beta diversity was carried out using the Bray–Curtis algorithm and Jaccard algorithm index. The Bray–Curtis algorithm revealed that the *B. velezensis* VB7 + *T. koningiopsis* TK + RKN had different communities of mycobiome that reside in tomato rhizosphere compared to the uninoculated control. The Jaccard algorithm index indicated that the diversity and similarity among the organisms varied for each sample at every taxonomic level. The fungal communities that reside in the rhizosphere soil had greater diversity with lesser homogeneity between populations in treated soils than in untreated soil (Figure 6).

### 3.7. Co-Occurrence Clustering Coefficient Analysis of Fungal Communities in Treated and Untreated Soil Samples

**Figure 7:**
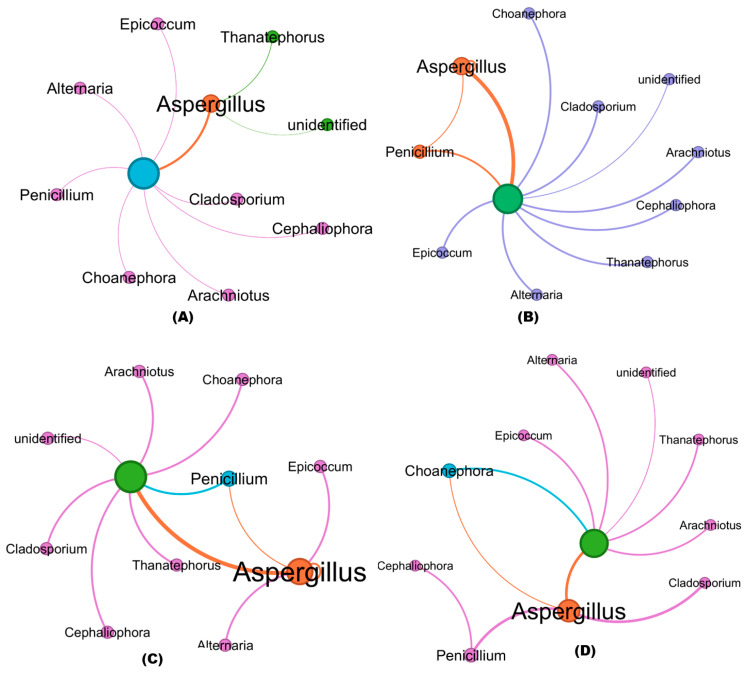

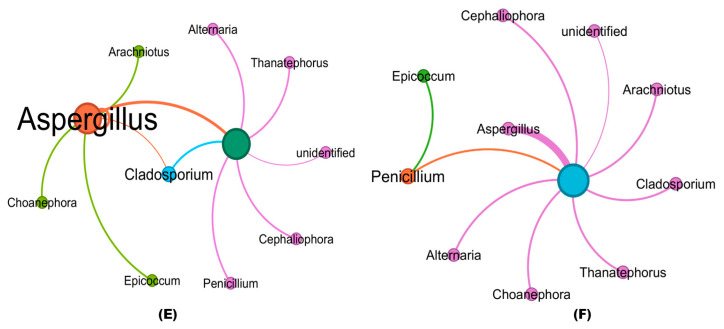
Co-occurrence clustering coefficient networks in datasets obtained from treated and RKN soil: (**A**) fungal communities in RKN-infected soil alone; (**B**) bacterial communities in the *T. koningiopsis* TK-applied soil; (**C**) bacterial communities in *T. koningiopsis* TK + RKN-treated soil; (**D**) bacterial communities in *B. velezensis* VB7 alone-treated soil; (**E**) bacterial communities in *B. velezensis* VB7 + RKN-treated soil; (**F**) bacterial communities in *B. velezensis* VB7 + *T. koningiopsis* TK + RKN-treated soil. Each node represents a bacterial phylum, whereas the edges represent a clustering coefficient, with a magnitude of 0.01 to 1.00 between the nodes. Each node is labeled at the genus level. The thickness of the edges represents the strength of clustering and interaction of fungal genus. Thicker edges with a boldness of fungal genera indicate greater clustering with strong interaction among fungal communities. The orange nodes and edges represent the interaction and co-occurrence of *Aspergillus*; violet nodes and edges represent the interaction and co-occurrence of other fungal genera.

The co-occurrence patterns of all networks differed significantly among treated and untreated RKN-infected soil samples. Co-occurrence network analysis revealed similar nodes (Phylum) among the fungi in the soil communities obtained from the bioagent-treated samples and untreated soil samples (Figure 7A–F). The numbers of nodes and their interconnecting edges (lines), however, differed. Among the fungal phyla, Ascomycota, Basidiomycota, and Mucoromycota were clustered in greater proportion with strong interaction in all the soil samples. The fungal genera including *Aspergillus*, *Penicillium*, *Cladosporium*, *Cephaliophora*, *Arachniotus*, *Epicoccum*, *Choanephora*, *Alternaria*, and *Thanatephorus* were mostly distributed in both treated and untreated soils. The nodes of Aspergillus interconnecting with other fungal genus edges are highlighted in orange, whereas the interconnections of other fungal genera are represented in violet and blue (nodes and edges). The strong correlation between the abundance of fungal genera (thicker lines) with a higher number of interconnection edges was greater in Aspergillus with the maximum proportion than other fungal genera, while Penicillium (orange) and *Epicoccum* (green) are clustered in open triplet edges in RKN-associated soils treated with *B. velezensis* VB7 + *T. koningiopsis* TK (Figure 7F). Hitherto, the interaction of *T. koningiopsis* TK in soils associated with RKN had the maximum interaction compared to *T. koningiopsis* TK alone without RKN (Figure 7B,C). The *B. velezensis* VB7 + RKN and *B. velezensis* VB7 alone-treated soil have sparse edges with a lesser coefficient witnessed with strong interaction compared to RKN samples (Figure 7D,E). The RKN soil without any bioagents applied has diverse thinner interconnecting edges (lesser) between fungal communities (Figure 7A). Collectively, the data provide further evidence that the fungal community in tomato rhizosphere soil was increased by the application of consortia comprising *B. velezensis* VB7 or *T. koningiopsis* TK, exhibiting a significant increase in the relative abundance of bacterial communities due to synergistic interactions.

## 4. Discussion

In the present investigation, we analyzed the antinemic potential of *B. velezensis* VB7 and *T. koningiopsis* TK for the management of root-knot nematode and tomato growth promotion. Furthermore, the diversity and richness of the mycobiome in bioagent-treated soils challenged with RKN and untreated rhizosphere soil samples in tomato plants were analyzed in field conditions.

Potential of the pathogens to infect crops would be suppressed more effectively and economically through biological control in protected agriculture. In the present study, soil drenching with combined application of *B. velezensis* VB7 and *T. koningiopsis* TK challenged with RKN at 15, 30, and 45 days after transplanting had the minimum gall index of 1.27 pertaining to root-knot nematode infection. Previous studies established the significance of *Bacillus* spp. [28,29,30] and *Trichoderma* spp. [31,32,33,34,35] as potential biocontrol agents for the control of root-knot nematode. A similar report [36] revealed that the tomato root drenched with *B. velezensis*-DS1 decreased the number of galls and egg masses by 29.3% and 33.8%, respectively. Eggs per egg mass from the Bv-DS1-drenched root system in the soil was also lower, compared to uninoculated plants. These findings suggested that Bv-DS1 might improve the antinemic ability to reduce the infestation of *M. incognita*. In another study, pot culture experiments revealed that the plants drenched with *B. velezensis*-25 reduced the disease severity index by 73.8% and increased the yield up to 11.35 t/ha [37]. Application of *T. harzianum* reduced the population of *M. javanica* J2s and the severity of Fusarium wilt in tomato crops [38]. The gel formulation of *T. koningiopsis* TRI 41 effectively suppressed fungal nematode complex and enhanced cucumber plant growth, reduced RKN complex to 13.45% with 80.0 fruits/plant and a mean yield of 15.01 t/1000 m^2^ [39]. The number of root galls, J2s, nematode egg masses, and J2s population density in soil were substantially decreased over 50% by the application of *T. citrinoviride* Snef1910 to tomato and significantly increased plant growth in greenhouse conditions [31]. Soils treated with *T. harzianum* and *T. viride* reduced the galls produced by RKN in tomato up to 30.8% [40].

With the advancement and application of molecular technology, the underlying mechanisms of soil suppression have been described in an important way. Naturally, tomato plant roots have a strong association with diverse microbial communities that reside in soil [41]. These microbiotas are part of a unique microbial community that aids in the growth and development of tomato plants. Understanding the mechanisms of a candidate microbiome that improves plant health is necessary for the microbiome’s efficiency in the rhizosphere [42]. Several investigations have demonstrated that the combined use of beneficial microorganisms can be effectively and successfully employed to activate the defense mechanisms against PPN [43]. Similarly, from the present investigation, the metagenomics results confirm the increased relative abundance of fungal biome in the tomato rhizosphere soil drenched with the consortia of *B. velezensis* VB7 + *T. koningiopsis* TK in the presence of RKN when compared to soils infested with RKN alone. The diverse mycobiome may have contributed to defend the parasitism of RKN and may have suppressed its pathogenicity. Several researchers have also confirmed that RKN parasitism can have a considerable impact on the richness and diversity of microbial communities associated with the rhizosphere soil microbiota [44,45,46,47]. Further, biocontrol agents can have a significant impact on the composition and diversity of the soil microbial communities, enhance the soil microecological environment, and reduce the parasitism of RKN occurrence [48,49]. As the taxonomic diversity of bioagents increases, they augment the diversity of secondary metabolites with broad-spectrum action associated with the suppression of plant parasitic nematodes and pathogens [50]. Furthermore, fungal community structure and diversity have been attributed to the suppression of RKN [51,52,53,54]. Likewise, variability in the suppressiveness of RKN may be due to the varied soil microbiome that inhabits the soil [55].

The most effective approach to prevent and control RKN is to regulate the structure of the rhizosphere microbial community and to maintain soil health. The structure of the rhizosphere microbial community is altered due to the introduction of bioagents. Synergistic interactions between introduced bioagents and their ability to enhance their performance through various mechanisms may contribute to a diverse and resilient microbial community, thereby benefiting the plant by stimulating growth and avoiding the pathogenic nematode attack. Further, few investigations have revealed that certain rhizosphere microbial groups contribute a significant part in facilitating the occurrence of RKN. It is feasible to reduce the prevalence and severity of RKN by comprehending and regulating these microbial communities.

In our study, we found that the composition and diversity of the microbiome varied with respect to treatments. The fungal phyla Ascomycota, Mucoromycota, and Basidiomycota were observed in all rhizosphere soils. Among them, Ascomycota was more predominant, with 92.68% composition compared to other phyla. Hitherto, the soils treated with *B. velezensis* VB7 + RKN, *T. koningiopsis* + RKN, and *B. velezensis* VB7 + *T. koningiopsis* + RKN had 100% abundance of Ascomycota population, except for *B. velezensis* VB7 alone and RKN-infested untreated soil (lower abundance). Huang et al. (2020) obtained similar outcomes in their investigations on the impact of biocontrol agents in structuring the soil microbial communities. According to their findings, Ascomycota was the most prevalent fungal phylum in both bioagent-treated and untreated soil samples, followed by Basidiomycota, which was 91% of the overall abundance. Our result corroborates with recent research on similar phyla obtained in the rhizosphere soil of tomato and other crops [56,57]. The aforementioned phylum of fungi developed mutualistic interactions with plants and was exploited as a biological weapon against various plant pathogens [58]. It is essential to comprehend that the microbial population in the soil is complex and diverse, and that it can be regulated by a wide range of factors. The total microbial species diversity was not significantly different at the phylum level since the architecture of the soil microbial community was not substantially different [59]. The overall diversity and richness of Ascomycota phylum in biocontrol-agent-drenched soil increased after treatment, while it was not present in untreated soil. Consequently, the presence of Ascomycota might prevent RKN proliferation and infection. Further, augmentation with the large number of microorganisms in the soil may alter the hierarchical structure of the regional microbial community and contribute to the expression of different traits [25].

Comparison of the relative abundance of fungal genera indicated that the abundance of *Aspergillus* (46.34%) and *Epicoccum* (9.75%) was more dominant than other genera. The abundance of Aspergillus genera in RKN soil treated with *T. koningiopsis* TK and *B. velezensis* VB7 had constituted 35.87% and 65.81% of relative abundance. However, it was not observed in untreated control. *Aspergillus* spp. is a filamentous fungus, known to have various biological activities, including the production of nematicidal compounds. It is also considered as a nematode-trapping fungus, which parasitizes the PPN by hyphal ring entrapment, followed by penetration and digestion of nematodes [60]. In several scientific reports, various Aspergillus species have putative antinemic properties against *Meloidogyne* spp., including *A. niger* [61], *A. welwitschiae* [62,63], *A. terreus*, and *A. japonicus* [64,65]. Palmitic acid, dodecanoic acid, oleic acid, linoleic acid, and quinazolinone are known to be produced by Aspergillus sp., and thus explored for their potential nematicidal activity [66,67].

Similarly, the genus Penicillium was also observed in the range 25.67% to 17.83% in the rhizosphere soils treated with bioagents. Investigation by different researchers worldwide has revealed that certain agricultural practices increase the population density of *Penicillium, Acremonium,* and *Chaetomium* genera and majorly contribute to the suppression of Fusarium wilt under field conditions [68,69,70,71]. In the present investigation, individual application of either bioagent, *T. koningiopsis* TK or *B. velezensis* VB7, induced the proliferation of *Epiccocum* spp.

Thus, augmentation of biocontrol agents to soil may have a positive influence on the abundance and diversity of the mycobiome. Similarly, the application of *T. harzianum* ESALQ and *T. asperellum* BRM changed the native soil microbial community composition and the endophytic community composition in the leaves and roots of dry bean [72]. The nematode-infested soil had a prevalence of *Thanatephorus* with 20% RA. Plants with sedentary endoparasitic nematode infestations induced the secretion of root exudate, which is beneficial to pathogen growth and its severity, and thus leads to complex diseases [73]. Similarly, concurrent infections with *Meloidogyne incognita* increased the severity of *R. solani* (*Thanatephorus* spp.) root rot in tomato [74]. 

We analyzed the diversity indices for the mycobiome in both treated and untreated soil. The results indicated that the alpha diversity index showed a higher microbiome abundance and diversity following the application of consortia-based bioagents challenged against RKN in tomato, when compared to untreated control. The beta diversity analysis revealed that diversity in the root microbiome was significantly different for each soil, and the heterogeneity among them was substantially greater between treated and nematode-infected roots. The soil treated with combined and individual application of bioagents had greater diversity and richness of microbial population than RKN-infested soil. Our results are in line with the findings in [15]. The heterogeneity of microbial communities observed in the soil sample suggested that the microbial metagenomes aid in identifying the precise roles of the microbial communities in the rhizosphere soil. This indicates that the application of bioagents may have positive influence on the diversity of the mycobiome. However, the addition of bioagents did not affect the richness and diversity of the endophytic, rhizosphere, and edaphic communities, but changed their composition [75]. In brief, by triggering the defense mechanism, the increased fungal species in the treated soil can serve as an alternative for the suppression of RKN infestation.

Combined application of *P. lilacinum, T. viride*, and *P. fluorescens* increased the efficiency of cucumber plants to defend against RKN infestation, which may be due to higher colonization of bioagents. The reduction in the severity of RKN infestation could be attributed to the combined actions of all bioagents [76]. Several researchers proved that the combined application of bioagents was effective in the management of *M. incognita* [77,78]. With the evidence of this statement, our finding also indicated the increased diversity of fungal population in the rhizosphere region may contribute to the suppression of RKN infection in tomato.

Subsequently, several investigations have already confirmed that colonization of plant roots by *Trichoderma* spp. enhanced the defense mechanism against nematodes. The biotic pressure on the microbial community assemblage improves plant endurance and increases microbial diversity [79], or causes dysbiosis, which triggers an abnormal increase in some minor taxa responsible for the suppression of RKN [80]. Likewise, infestation of root-knot nematode changed the composition, function, network, and metabolic activity of soil microorganisms in the rhizosphere [81]. Hence, the diverse community of microorganisms that inhabit the soil around plant roots can play an important role in helping plants to defend themselves against plant-parasitic nematodes.

Enhancing the diversity and abundance of helpful microbes by modifying the soil ecosystem with bioagents and nematicide mitigates the significant damage inflicted by nematode infestations and is considered as an effective approach for RKN management. Since the structure of microbiome community is the first line of defense, our study will inevitably serve as a baseline for additional investigation toward developing a microbiome-based management technique for RKN, which poses an imminent threat to tomato production.

In summary, the consortia of bioagents and nematicides may enrich the microbiome communities and enhance the biological activities of microorganisms that reside in the rhizosphere region, and thus suppress the RKN infestation. This is because applied bioagents and associated microorganisms can help to reduce the population of nematodes by parasitizing them or inducing plant resistance, while bioagents can provide immediate control of the nematodes and reduce the risk of developing resistance at different stages of the nematode’s lifecycle by different modes of action. The findings provide a glimpse into the antinemic activity of bioagents by altering the structure of soil microbiome, particularly of those associated with the tomato rhizospheres.

## 5. Conclusions

We investigated the effects of microbial diversity from bioagent-treated tomato plants through high-throughput sequencing analysis. Our results revealed that combined application of bioagents challenged against RKN had the maximum relative abundance in the microbial community. The fungal phylum Ascomycota was dominant and had the higher relative abundance with increased frequency of unique clustering of OTUs. The soil applied with bioagents *B. velezensis* VB7 and *T. koningiopsis* TK in association with RKN had the maximum diversity and relative abundance in biologically active microbial genera comprising *Aspergillus* and *Penicillium*. It may have served as a bionematicide against RKN and enhanced plant growth. Overall, our results indicated that the combined application of bioagents altered the composition of the community structure of the fungal biome in the rhizosphere, leading to the diverse accumulation of antinemic biomolecules and induction of defense mechanism responsible for the suppression of nematode infection, in addition to promoting the plant growth.

Further, our findings also provide critical information for establishing long-term agricultural management methods for reducing RKN infestations by amending the soil with beneficial microbial community, which would suppress nematode infestation and promote plant health. To provide prescriptive management for sustainable agriculture, diversity in community structure of fungal biome will definitely establish the way for sustainable management of RKN rather than the application of an individual antagonist.

## Data Availability

The original contributions presented in the study are included in the article/Supplementary Materials; further inquiries can be directed to the corresponding author.

## Supplementary Materials

The following supporting information can be downloaded at: https://www.mdpi.com/article/10.3390/microorganisms11102467/s1.
