# Supplementary material for: Metagenomic Approach Deciphers the Role of Community Composition of Mycobiome Structured by Bacillus velezensis VB7 and Trichoderma koningiopsis TK in Tomato Rhizosphere to Suppress Root-Knot Nematode Infecting Tomato
Source: Microorganisms. 2023 Sep 30;11(10):2467. doi: 10.3390/microorganisms11102467 (PMC10609121; doi:10.3390/microorganisms11102467)
Supplement: Supplementary file 1 [file microorganisms-11-02467-s001.zip › microorganisms-2630300-supplementary.pdf]

Supplementary Information

Title for table

Table S1. Effect of the liquid formulation of *B. velezensis* VB7 and *T. koningiopsis* TK against Root Knot Nematode (*M. incognita*) in tomato under greenhouse conditions.

TREATMENTS	Plant height at 30 DAT (cm)	Plant height at 50 DAT (cm)	Plant height at 65 DAT (cm)	Root gall index	Fruit weight g/fruit	Yield kg/plant
**T_1 - *B. velezensis* VB7 (1 %) + RKN**	55.19^{b}	84.73^{b}	119.67^{b}	2.13^{c}	114.60^{b}	1.52^{b}
**T_2 - *B. velezensis* (1 %)**	52.67^{b}	80.46^{c}	115.89^{b}	0.0^{a}	110.30^{c}	1.43^{c}
**T_3 - *Trichoderma koningiopsis* (1 %) + RKN**	49.50^{c}	76.46^{d}	110.34^{c}	2.56^{d}	104.58^{d}	1.15^{d}
**T_4 - *Trichoderma koningiopsis* (1 %)**	45.70^{d}	71.17^{e}	104.73^{d}	0.0^{a}	100.46^{e}	1.07^{e}
**T_5 - *B. velezensis*(1 %) + *Trichoderma koningiopsis* (1 %) + RKN**	62.40^{a}	90.10^{a}	127.45^{a}	1.27^{b}	120.75^{a}	1.74^{a}
**T_6 - RKN**	30.61^{e}	52.82^{f}	85.68^{e}	4.0^{e}	80.64^{f}	0.5^{f}
**CD(0.05)**	1.758	3.977	3.936	0.093	3.973	0.073

Values are the means of three replications.In a column means followed by a common letter are not significantly different at the 5% level by DMRT and Values in parenthesis are arc sine transformed values.

Title for supplementary Figures S1-S6 (Composition of the fungal communities in different treatments)

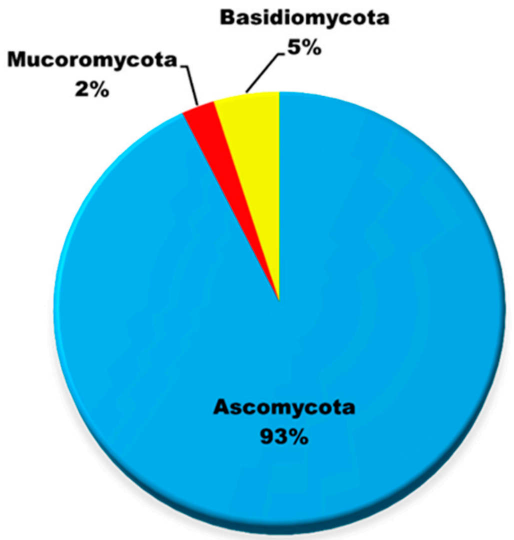

Figure S1. Composition of fungal phyla with respect to different treatments.

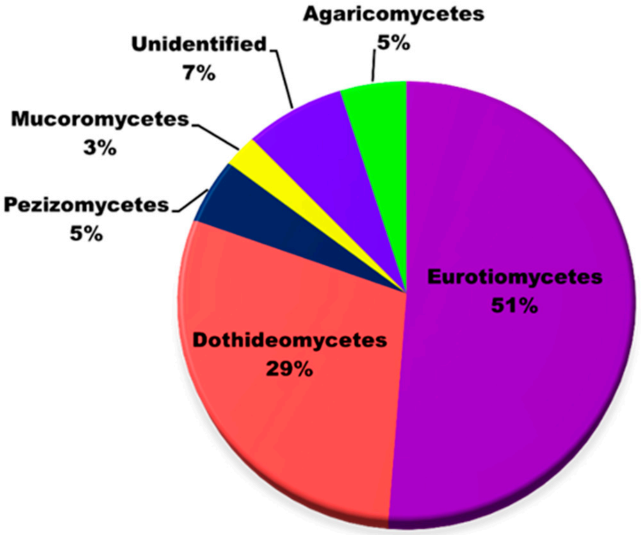

Figure S2. Composition of fungal classes with respect to different treatments.

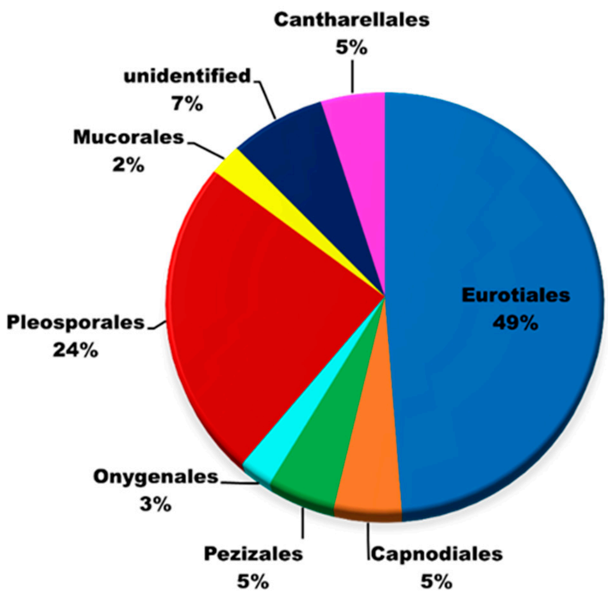

Figure S3. Composition of fungal orders with respect to different treatments.

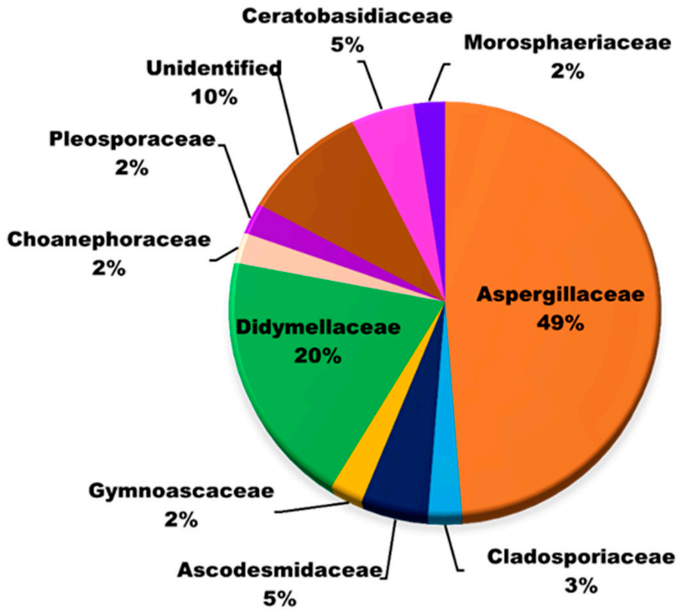

Figure S4. Composition of fungal families with respect to different treatments.

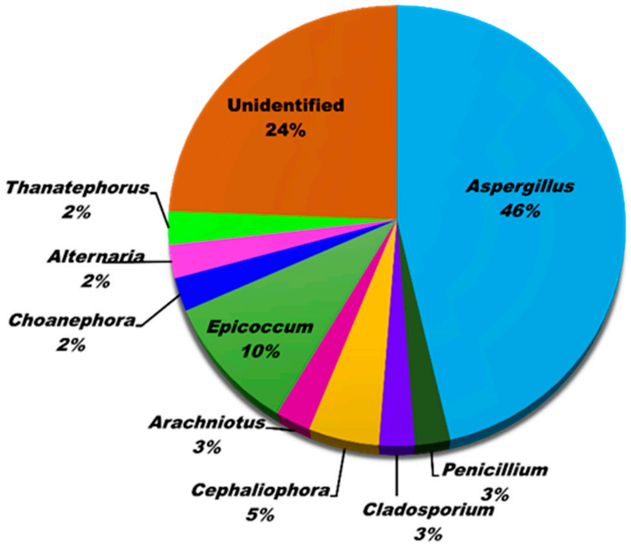

Figure S5. Composition of fungal genera with respect to different treatment.

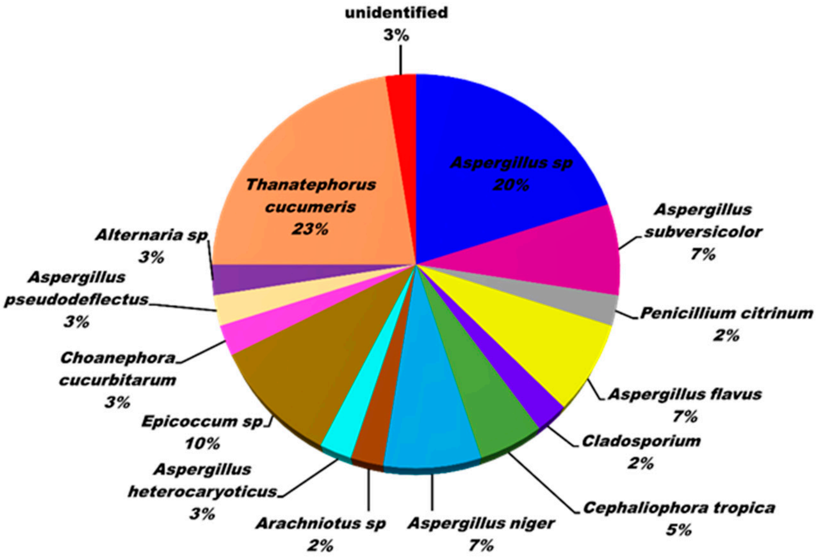

Figure S6. Composition of fungal species with respect to different treatment
